# Impact of 2D versus 3D fibroblast models on *Leishmania* species invasion *in vitro*: Rab5 dynamics and actin activity in initial infection

**DOI:** 10.3389/fcimb.2025.1654654

**Published:** 2025-08-18

**Authors:** Rebecca Thereza Silva Santa Brígida, Adeniele Lopes da Cruz Carneiro, Felipe Tuji de Castro Franco, Brenda Furtado Costa, Ana Paula Drummond Rodrigues

**Affiliations:** ^1^ Multiuser Laboratory of Cell Biology and Ultrastructure, Evandro Chagas Institute, Belém, Pará, Brazil; ^2^ Postgraduate Program in Biology of Infectious and Parasitic Agents, Federal University of Pará, Belém, Pará, Brazil; ^3^ Postgraduate Program in Parasitic Biology of the Amazon, University of State of Pará, Belém, Pará, Brazil

**Keywords:** *Leishmania*, fibroblasts, 3D cell culture, Rab5, actin cytoskeleton, parasite invasion

## Abstract

**Background:**

The protozoan *Leishmania*, in addition to infecting phagocytic cells such as macrophages, can also invade non-professional phagocytic cells like fibroblasts, a process previously described in 2D models. In a bidimensional environment, its interaction with the extracellular matrix and manipulation of endocytic processes reveal a complex ability to alter cellular entry mechanisms. However, this process in fibroblasts, especially in three-dimensional (3D) models, remains poorly understood. *In vitro* 3D models more accurately replicate the cellular microenvironment under physiological conditions. This study is the first to investigate the initial infection process of *L. (L.) amazonensis* and *L. (V.) braziliensis* in murine fibroblasts using a 3D model, with a comparative analysis to the 2D model.

**Methods:**

3T3 fibroblasts were exposed to promastigotes of both *Leishmania* species for 5, 18, and 24 hours in 2D (plate coverslips) and 3D (type I collagen matrix) models. The infection was analyzed using immunofluorescence and confocal microscopy, which evaluated the adhesion index, actin involvement, and Rab5 recruitment—an early endosomal marker.

**Results:**

Higher adhesion of *L. amazonensis* was observed in 2D, while *L. braziliensis* adhered more in 3D. Membrane protrusions (filopodia and lamellipodia) were seen near the parasites, indicating cytoskeletal activity. Rab5 was strongly recruited around *L. amazonensis* in the 3D model, whereas its labeling was weak in the control groups and the *L. braziliensis* 3D group. In the 2D model, Rab5 labelling was more pronounced in both infected groups. Throughout the interaction periods, Rab5 played a more prominent role in the entry of *L. amazonensis*, suggesting that actin’s secondary participation was involved. In contrast, *L. braziliensis* appeared to rely more heavily on actin-dependent entry routes, particularly at 24 hours.

**Conclusions:**

These novel findings reveal that distinct Leishmania species utilize specialized invasion strategies, adapting to both host cell type and experimental conditions. This underscores the role of species-specific biological traits in modulating host cell entry mechanisms, which may, in turn, influence the varied clinical manifestations associated with each species.

## 1 Introduction

Cutaneous leishmaniasis is an infection caused by protozoa of the genus *Leishmania*, with an estimated 0.6 to 1.0 million new cases occurring worldwide each year (World Health Organization, 2023). In South America, *Leishmania (Leishmania) amazonensis* and *Leishmania (Viannia) braziliensis* are among the primary species responsible for a wide range of clinical manifestations, ranging from single ulcers to severe lesions (Burza et al., 2018).

These variable in cutaneous manifestations result from the different host immune responses directed by these different species (Scorza et al., 2017). Infection with *Leishmania amazonensis* often results in a hyposensitivity pole response characterized by a predominant Th2-type immune response, resulting in a diffuse anergic form. In contrast, *Leishmania braziliensis* typically triggers a hypersensitivity response with an exacerbated Th1-type response, leading to more severe forms, such as mucocutaneous leishmaniasis (Silveira et al., 2009). The infection in the mammalian host begins in the skin when female sandflies inoculate metacyclic promastigotes into this tissue. The skin, and more specifically its dermal layer, is primarily composed of extracellular matrix and fibroblasts (Chambers and Vukmanovic-Stejic, 2020). The extracellular matrix is a three-dimensional dynamic network that provides support to cells, composed of macromolecules such as fibrillar proteins (collagen, fibronectin, elastin, laminins) and non-fibrillar constituents (proteoglycans, glycosaminoglycans, and glycoproteins) (Theocharis et al., 2016, 2019). Therefore, the extracellular matrix is the first and most important obstacle that the parasite needs to overcome/interact with for the establishment of the infection (De Menezes et al., 2016).

In the skin, the initial infection process is marked by the capture of the metacyclic promastigotes by phagocytic immune cells such as neutrophils and later macrophages, where they assume their intracellular amastigote form (Teixeira et al., 2013; Conceição-Silva and Morgado, 2019). In the process of endocytosis, such as phagocytosis, the target is engulfed by membrane protrusions formed from the rearrangement of the actin cytoskeleton (Mylvaganam et al., 2021). This engulfed target then fuses with membranous compartments called the endosomes, which will undergo a maturation process where early endosomes are converted into late endosomes. In this final stage, they can fuse with lysosomes, leading to the degradation of their content (Scott et al., 2014; Levin et al., 2016) However, the *Leishmania* parasite can manipulate these mechanisms to ensure its survival within these cells (Scianimanico et al., 1999; Desjardins and Descoteaux, 1997; Dermine et al., 2005).

Although the 2D cell culture model is still widely used due to its simpler execution, good reproducibility and lower cost (Costa et al., 2016), 3D cell culture better mimics the biochemical, mechanical, and spatial characteristics of the microenvironment in an *in vivo* model (Souza and Ferreira, 2016; Duval et al., 2017) Various types of 3D models can be used (e.g. scaffold, spheroids, etc.) (Costa et al., 2016); however, the extracellular matrix, as the model used in this study, can imitate cell-extracellular matrix interactions and mechanical properties like those found in natural environments (Slaughter et al., 2009; Breslin and O’Driscoll, 2013).

In fibroblasts, although the ability of the Leishmania parasite to infect fibroblasts is known, the infection process involving cellular mechanisms remains poorly understood, with few studies conducted in 2D models (Cavalcante-Costa et al., 2019). Moreover, the comparative behavior of different *Leishmania* species during fibroblast infection remains unexplored, despite their known clinical and immunological differences (Silveira et al., 2009). In 3D models, however, there is still no description of the initial infection process of *Leishmania* involving fibroblasts. This gap is particularly relevant since three-dimensional environments can influence host cell behavior, parasite access, and pathways that may alter the infection outcome.

Although actin remodeling is central to parasite entry in phagocytes, how *Leishmania* interacts with the actin cytoskeleton in non-phagocytic cells like fibroblasts - especially under 3D conditions - remains unclear. Notably, *L. amazonensis* can invade fibroblasts via a non-canonical, actin-independent pathway involving calcium signaling and lysosome-mediated membrane repair (Cavalcante-Costa et al., 2019). These findings indicate that Leishmania is capable of exploiting alternative entry pathways in non-professional phagocytes. However, the extent to which such mechanisms are preserved or modulated under more physiologically relevant 3D conditions has yet to be elucidated.

Additionally, although Rab5 is a representative member of the GTPase family involved in vesicular trafficking and is a critical early endosomal marker required for the formation of early endosomes (Stenmark, 2009), its recruitment and role during *Leishmania* entry into fibroblasts have not yet been investigated. Elucidating whether Rab5 participates in alternative, actin-independent entry pathways may provide important insights into how the parasite traffics and survives within non-phagocytic host cells.

Together, these knowledge gaps underscore the need for a deeper understanding of *Leishmania* infection in fibroblasts and for the identification of potential mechanisms by which the parasite adapts to and manipulates non-professional phagocytic host cells. Accordingly, we hypothesize that *L. amazonensis* and *L. braziliensis* differ in their early modes of fibroblast invasion, as reflected by distinct Rab5 dynamics and actin remodeling under 2D and 3D *in vitro* conditions.

## 2 Materials and methods

### 2.1 Parasite culture

The promastigote forms of *Leishmania (Leishmania) amazonensis* (MHOM/BR/26361) and *Leishmania (Viannia) braziliensis* (MHOM/BR/M17323) were obtained from the Leishmaniasis Program at the Evandro Chagas Institute. The promastigotes were recovered from cryogenic vials, cultured in NNN medium, and maintained in RPMI 1640 medium (Roswell Park Memorial Institute), supplemented with fetal bovine serum (FBS), in a 27°C incubator. For the experiments, the promastigotes were cultured for seven days to reach the stationary phase. They were transferred to 15mL centrifuge tubes, centrifuged at 1500 RPM for 10 minutes, counted in the Neubauer chamber, and adjusted to a concentration of 2x10^6^ parasites per mL.

### 2.2 Murine cell culture

Murine fibroblasts (BALB/c 3T3, clone A31 - BCRJ:0047) from the Rio de Janeiro Cell Bank were cultured using the following procedure. The cells were grown in DMEM medium supplemented with 10% FBS and maintained until they reached 80% confluence. The cultures were washed with sterile phosphate-buffered saline (PBS) and subjected to trypsin 0.05% trypsin-EDTA for 3 minutes in an atmosphere of 5% CO_2_ at 37°C. To neutralize the trypsin, DMEM supplemented with 10% FBS was added. The cell suspensions were transferred to tubes and centrifuged at 1500 RPM for 5 at 4°C. The supernatants were discarded, and thexsedimented cells were resuspended in sterile PBS, followed by another centrifugation under the same conditions. The supernatants were discarded again, and the cells were resuspended in DMEM, counted using a Neubauer chamber, and adjusted to a concentration of 1x10^5^ cells per mL.

### 2.3 Extracellular matrix

To prepare the extracellular matrix, a conical microtube on ice was filled with 637 µl of type I collagen from rat tail (1.3 mg/ml, Gibco^®^), 735 µl of PBS, 63.7 µl of 10x DMEM culture medium, 63.7 µl of 10x RB reconstitution buffer (0.26 M NaHCO_3_, 0.2 M HEPES), and 15 µl of fibronectin (1 µg/100 µl), totaling 1.5 ml. For each well, 50 μl of this matrix gel was used to coat LabTek^®^ coverslip chambers. An additional 200 µl of the matrix gel was mixed with 3T3 fibroblasts and *Leishmania* species at a 1:20 cell-to-parasite ratio. After applying 50 µl of the collagen matrix gel to the chambers, the mixture (200 µl) was added and incubated at 37°C for 10 minutes to allow collagen polymerization. Another 50 µl of collagen matrix gel was then added on top of the polymerized mixture and incubated again under the same conditions. Following this, 500 µl of DMEM supplemented with 10% FBS was added per well and incubated for 5, 18, and 24 hours. After each incubation period, the supernatants were removed, and the cells were fixed for further experiments. Control groups without infection were maintained under identical conditions, and all experiments were conducted in duplicate.

### 2.4 Immunofluorescence

For analysis of the early endosome formation, cells were fixed with 3% paraformaldehyde, washed, and permeabilized with 3% PBS-BSA for 20 minutes. Aldehyde sites were blocked with 50 mM ammonium chloride for 40 minutes, followed by a second blocking step with 10% goat serum for 1 hour. Cells were incubated with primary rabbit anti-Rab5a. After incubation, the cells were washed with 3% BSA and 1% PBS-BSA (Phosphate buffer saline with bovine serum albumin and Tween) and then incubated for 45 minutes with secondary goat anti-rabbit antibody conjugated with Alexa 488. After washing, cells were incubated with phalloidin to label actin filaments and access actin-dependent endocytosis. Cell nuclei were stained with DAPI (1 mg/mL).

The experiment was visualized after 5h, 18h, and 24h of interaction on a Leica SP8 confocal microscope. 3D image acquisition and processing were conducted using the Leica Application Suite X software. The images were further processed in 3D, applying the mixed function, and compiled into the panel using Adobe Photoshop version 25.3.1.

### 2.5 Adhesion index

The adhesion index was based on counts of 100 fibroblasts in random fields, using images obtained from the immunofluorescence assay and the differential interference contrast on Leica SP8 confocal microscope (for 3D experiments) and the formula for determining the adhesion index, which considers the number of promastigotes adhered to the cell surface, was applied based on the infection rate (Elcicek et al., 2013).


$$\begin{array}{c} \text{Adhesion index}\\ =\;\frac{\text{\% cells with attached parasites }\times \text{Number of attached parasites}}{\text{Number of cells with attached parasites}} \end{array}$$

Three independent experiments were performed in three replicates (n = 3), and comparisons between groups were conducted using Student’s *t*-test, with differences considered statistically significant at *p* < 0.05.

## 3 Results

### 3.1 Distinct adhesion profiles of *Leishmania* in fibroblasts: 2D favors initial adherence, but *L. braziliensis* displays higher adhesion in 3D over time

**Figure 1:**
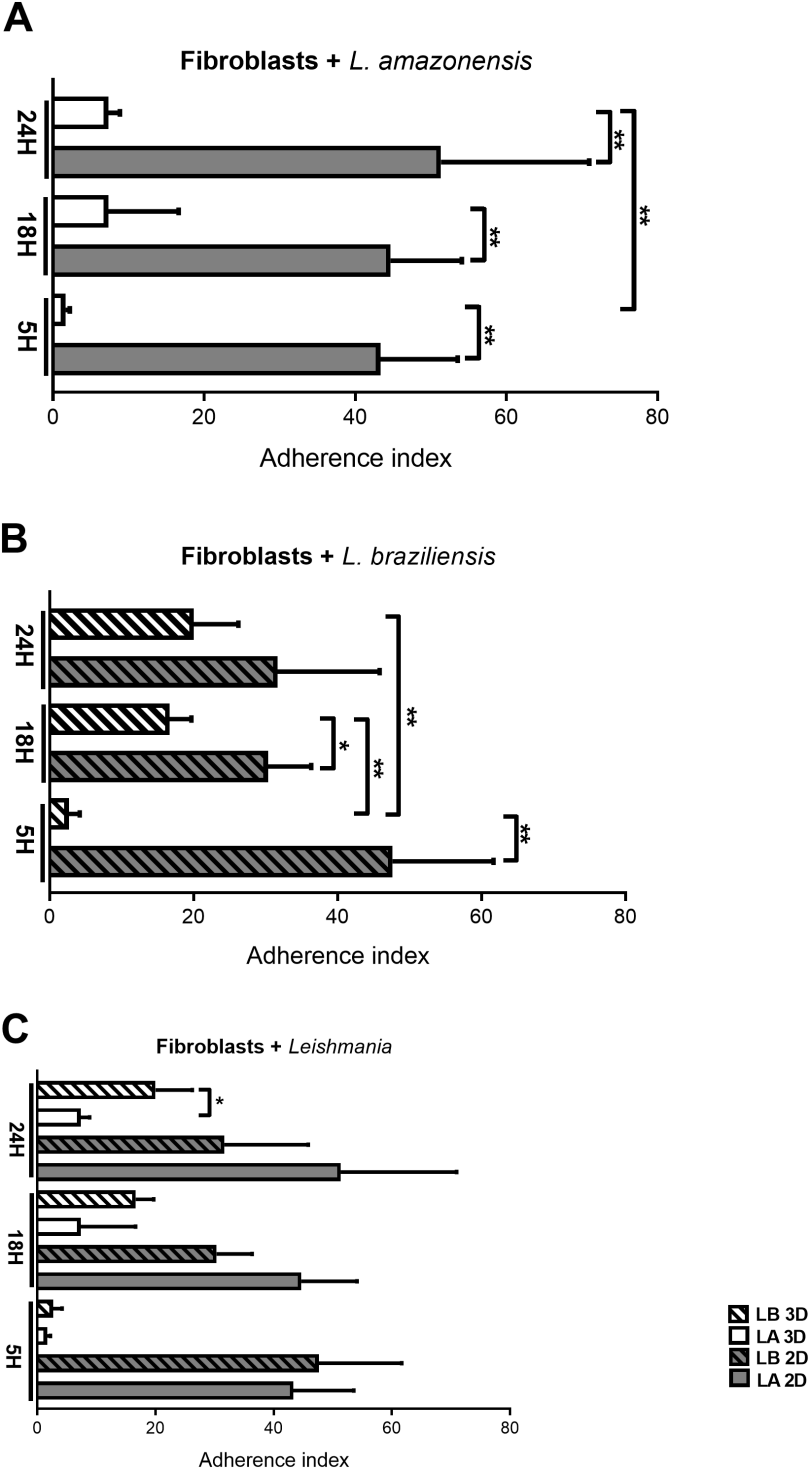
Adhesion index of Leishmania during fibroblast invasion at 5, 18, and 24 hours in 2D and 3D models. **(A)** Fibroblasts incubated with *L. amazonensis* comparing periods and models. **(B)** Fibroblasts with *L. braziliensis* comparing periods and models. **(C)** Fibroblasts in 2D and 3D models in different periods comparing *L. amazonensis* and *L. braziliensis* species adhesion. Student’s t-test is considered statistically significant when p < 0,05 (*), p < 0,01 (**).

The counting of attached parasites was performed at all interaction periods stipulated in the study (5h, 18h, and 24h). Overall, the count of *L. amazonensis* adhered to fibroblasts (*Fibroblasts + L. amazonensis*) demonstrated greater adherence in the 2D model compared to the 3D model over time (5h: p = 0.021; 18h: p = 0.0082; 24h: p = 0.0180). However, in the 3D model, there was a tendency for adherence to increase over time with a statistically significant difference at times 5h and 24h (p = 0.0039),which was not observed for the 2D model, with no significant statistical difference between these two periods (**Figure 1A**).

In the group of *L. braziliensis* incubated with fibroblasts (Fibroblast + *L. braziliensis –* **Figure 1B**), there was also greater adherence in the 2D model compared to the 3D model in the early periods (5h: p = 0.0052, 18h: p = 0.0248), however, in the later period (24h), no statistically significant difference was observed in the number of attached parasites. Furthermore, the 3D model showed a tendency to increase parasite adhesion over time (5h vs. 18h: p = 0.0021; 5h vs. 24h: p = 0.0095). On the other hand, in 2D there was no significant difference between the 3 periods of infection (**Figure 1B**). These trends in adherence index in the 2D model are similar to those observed in the *L. amazonensis* group (**Figure 1A**).

On the other hand, when we compared the adherence index between *Leishmania* species in fibroblasts (Fibroblasts + *Leishmania*), there was a significant increase in the adhesion index of *L. braziliensis* compared with *L. amazonensis* in the 3D model at 24h (p = 0.0270), indicating adhesion index only plays difference between species (with *L. braziliensis* adhering more than *L. amazonensis*) in one of the models (3D) at later period (24 h) (**Figure 1C**).

### 3.2 Actin remodeling in fibroblasts depends on the culture model and *Leishmania* species: 3D model limits projections while species dictate actin-parasite interaction

**Figure 2:**
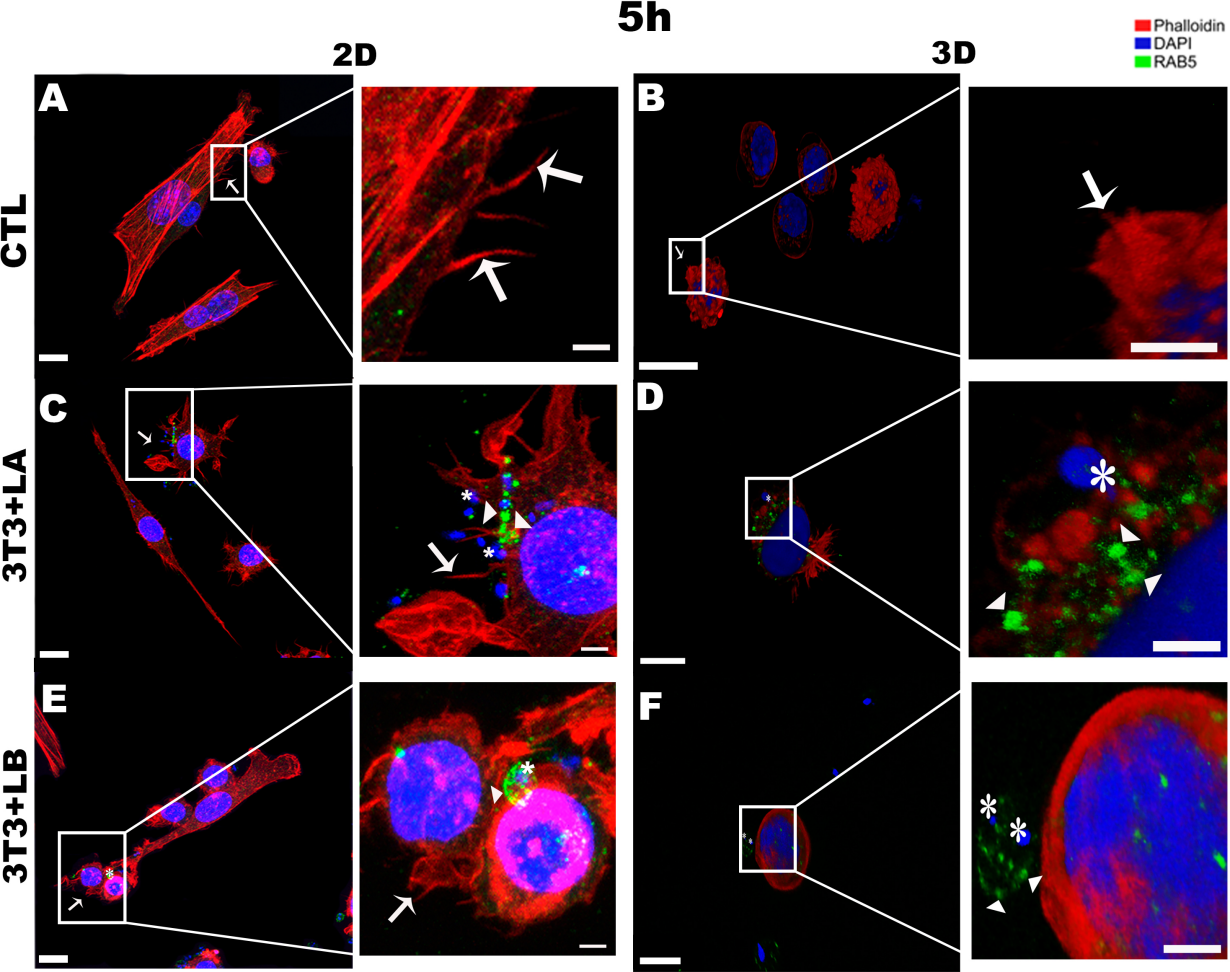
Immunofluorescence labeling for early endosome (Rab5 - arrowheads) and actin (Phalloidin^®^ - arrows) for 5 hours of interaction between fibroblasts and Leishmania species (asterisks) in 2D and 3D models. **(A)** CTL – Fibroblasts without infection in 2D. **(B)** CTL – Fibroblasts without infection in 3D. **(C)** 3T3+LA - Fibroblasts with *L. amazonensis* in 2D. **(D)** 3T3+LA - Fibroblasts with *L. amazonensis* in 3D. **(E)** 3T3+LB – Fibroblasts with *L. braziliensis* in 2D. **(F)** 3T3+LB - Fibroblasts with *L. braziliensis* in 3D. Scale bar = 10μm **(A, C–F)**, 20 μm **(B)**; Scale bars inserts = 10 μm (**A–C, E**, 5 μm **D, F**).

Regarding the actin cytoskeleton, at the earliest interaction period (5 hours), fibroblasts in the 2D model exhibited a higher spread-out cell cortex (**Figures 2A, C, E**). In contrast, in the 3D model, fibroblasts in all groups (control and infected) displayed retracted cortex (**Figures 2 B, D, F**). Furthermore, it was possible to observe the presence of projections formed from the rearrangement of the actin cytoskeleton, similar to filopodia (arrows), in the control group (**Figure 2A**) and the group infected with *L. amazonensis* in the 2D model, adjacent to the parasites (**Figure 2C** - asterisks). On the other hand, the *L. braziliensis* did not show the same pattern, as actin was observed around the parasite. (**Figure 2E** - asterisks).

**Figure 3:**
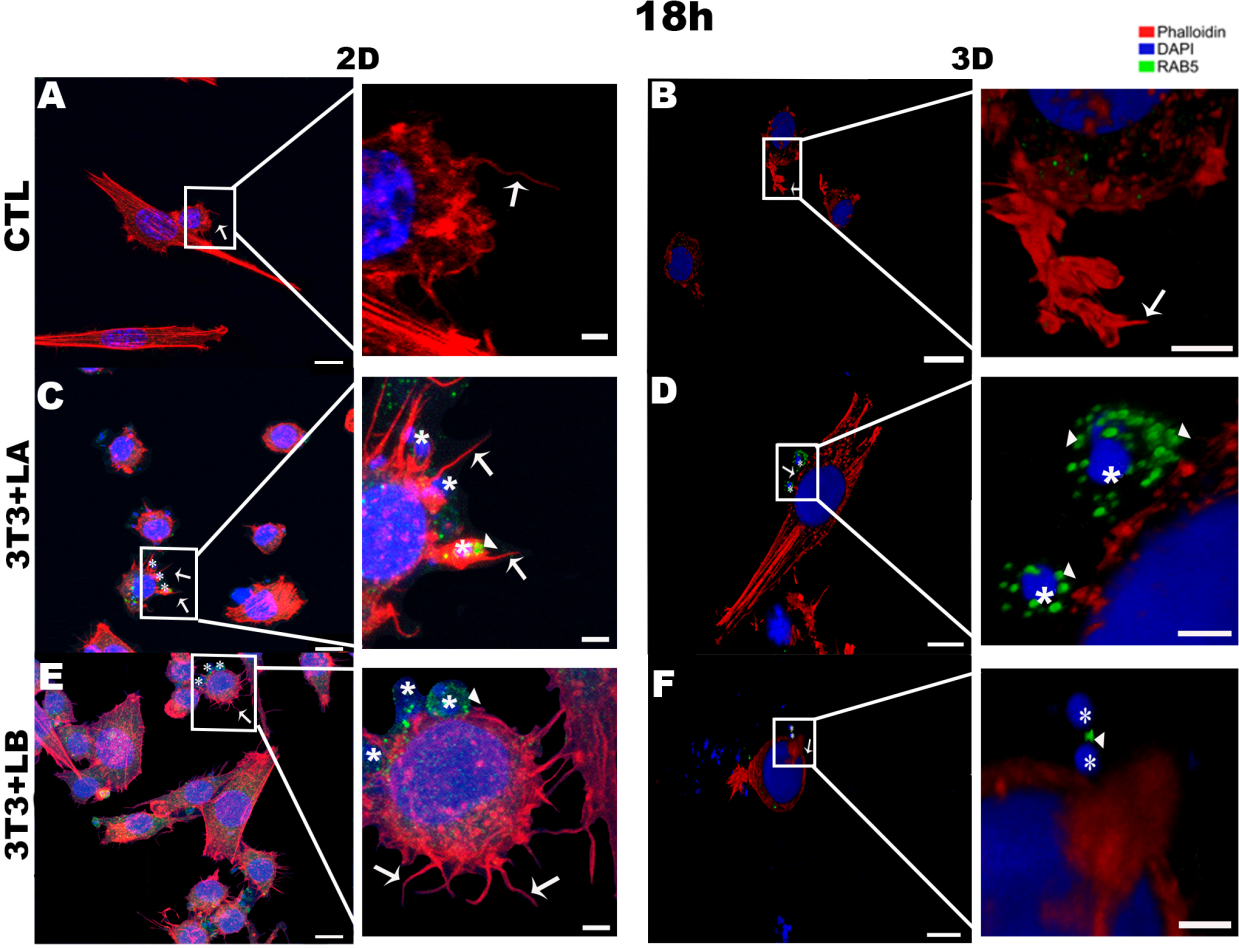
Immunofluorescence labeling for early endosome (Rab5 - arrowheads) and actin (Phalloidin^®^ - arrows) for 18 hours of interaction between fibroblasts and Leishmania species (asterisks) in 2D and 3D models. **(A)** CTL – Fibroblasts without infection in 2D. **(B)** CTL – Fibroblasts without infection in 3D. **(C)** 3T3+LA - Fibroblasts with *L. amazonensis* in 2D. **(D)** 3T3+LA - Fibroblasts with *L. amazonensis* in 3D. **(E)** 3T3+LB – Fibroblasts with *L. braziliensis* in 2D. **(F)** 3T3+LB - Fibroblasts with *L. braziliensis* in 3D. Scale bar = 10μm **(A, C–F)**, 20 μm **(B)**; Scale bars inserts = 10 μm **(A–D)** 5 μm **(E, F)**.

**Figure 4:**
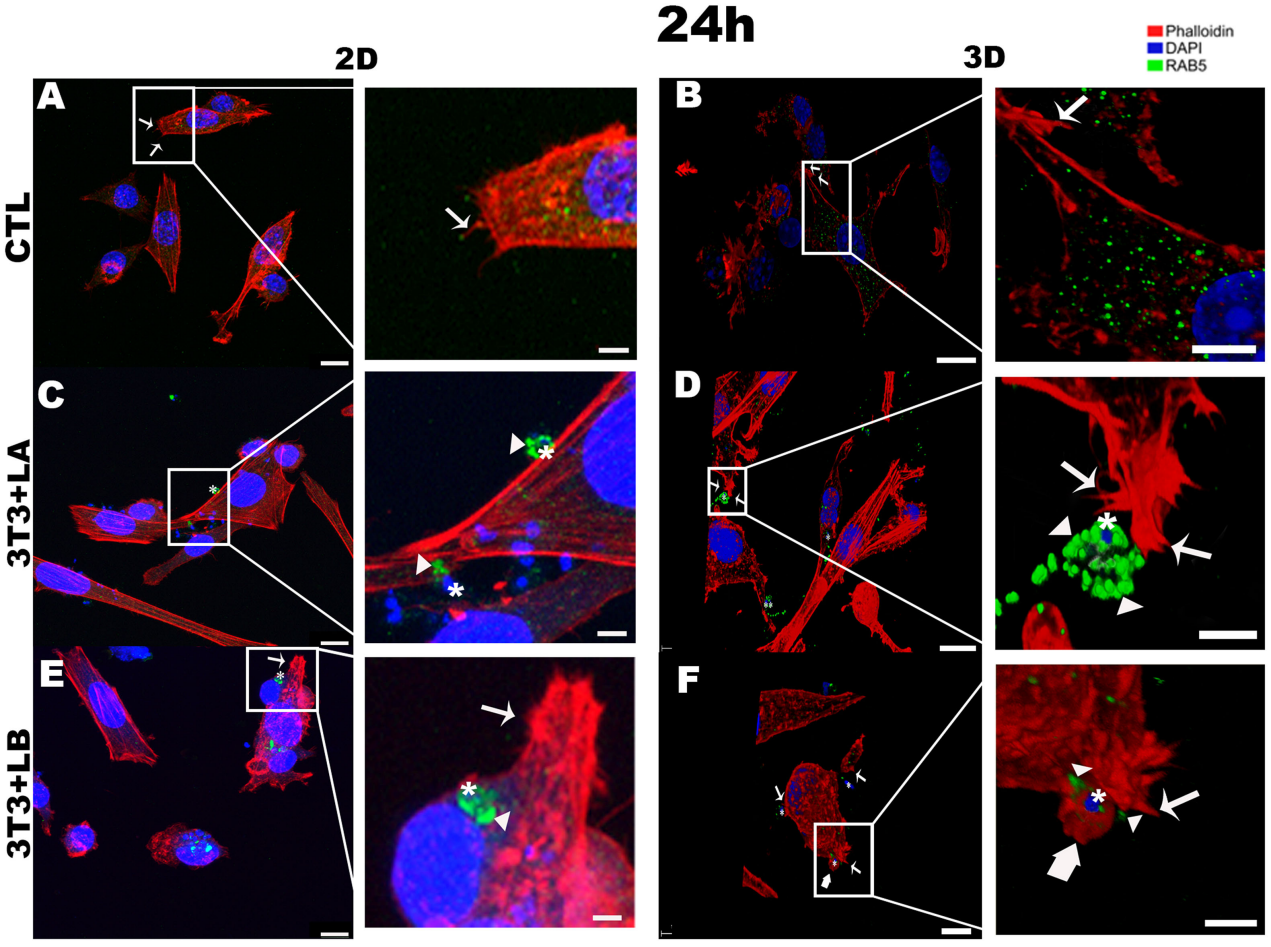
Immunofluorescence labeling for early endosome (Rab5 – arrowheads) and actin (Phalloidin^®^ - arrows) for 24 hours of interaction between fibroblasts and Leishmania species (asterisks) in 2D and 3D models. **(A)** CTL – Fibroblasts without infection in 2D. **(B)** CTL – Fibroblasts without infection in 3D. **(C)** 3T3+LA - Fibroblasts with *L. amazonensis* in 2D. **(D)** 3T3+LA - Fibroblasts with *L. amazonensis* in 3D. **(E)** 3T3+LB – Fibroblasts with *L. braziliensis* in 2D. **(F)** 3T3+LB - Fibroblasts with *L. braziliensis* in 3D. Scale bar = 10μm **(A, C, F)**, 20 μm **(B, D, E)**; Scale bars inserts = 10 μm **(A–D)**, 5 μm **(E, F)**.

In the 3D groups infected with different *Leishmania* species, no actin projections were observed near the parasites during the initial interaction period (5h) (**Figure 2D, F**). However, the actin cytoskeleton surrounded the *L. amazonensis* parasite (**Figure 2D**). After 18 and 24 hours of interaction, in the 3D model, it was possible to observe the actin cytoskeleton of the cells being widespread (**Figures 3**, **4**, respectively) compared to the 5 hours (**Figure 2**). In 2D, the group infected with *L. amazonensis* (**Figure 3C** - asterisks) *and L. braziliensis* (**Figure 3E** - asterisks). After 18 hours, it was possible to observe membrane projections that surround the parasite, resulting in shapes similar to a cup - actin cups - in addition to thin projections - filopodia (**Figures 3C, E** - arrows). In the 3D model, for both species, actin projections surrounding the parasites were observed less prominently (**Figures 3D, F**).

After 24 hours in the 2D model, parasites were observed inside host cells in the *L. braziliensis* group (**Figure 4E**), with actinaccumulating around the parasites (asterisk); however, this actin pattern was not observed in the *L. amazonensis* group (**Figure 4C** – asterisks). However, in the 3D model, filopodia formed from the rearrangement of the actin cytoskeleton (arrows) were observed in both *L. amazonensis*and *L. braziliensis*groups (**Figures 4D, F** –arrows, respectively), while lamellipodia were observed only in the *L. braziliensis* group (**Figure 4F** – large arrow).

### 3.3 Time-dependent recruitment of Rab5 during fibroblast infection reveals early involvement in *L. amazonensis* entry in 3D model

The Rab5 signal was qualitatively assessed by confocal microscopy based on the observation of fluorescence patterns, allowing comparison of labeling across different experimental conditions. At the 5-hour interaction, Rab5 labelling was not significantly observed in the control group for either the 2D or 3D models (**Figures 2A, B**, respectively). In the 3D model, Rab5a (arrowheads) appeared as discrete punctate signals throughout the cytoplasm but with weaker intensity, a pattern not observed in the 2D model (**Figures 2C, E** – arrowheads). In infected groups, Rab5 was detected near or surrounding the parasites in both models (**Figures 2D, F**).

At 18 hours of interaction, Rab5 appeared as discrete punctate signals and was weak in the 2D and 3D-control groups (**Figures 3A, B**, respectively). However, in both models, Rab5 was more prominently detected in the infected groups, particularly in the 3D-*L. amazonensis* group (**Figure 3D** - arrowheads, see **Supplementary Video 1**). The intensity around the parasite seems to be higher than 3D-*L. braziliensis* (**Figure 3F** – arrowheads) and 2D-*L. amazonensis* and *L. braziliensis* groups (**Figures 3E, F** - arrowheads, respectively).

At 24 hours, in the 3D model, Rab5 (arrowheads) followed a pattern similar to that observed at 18 hours, with stronger labelling surrounding the *L. amazonensis* parasite (**Figure 4D** - asterisks, see **Supplementary Video 2**) compared to the other groups. In contrast, Rab5 appeared as weak punctate signals in the cytoplasm of both the 2D and 3D control groups (**Figures 4A, B**) and Rab5 (arrowheads) was only faintly detected adjacent to the parasite in the 3D *L. braziliensis* (asterisks) group (**Figure 4F**). In the 2D model, however, Rab5 labelling was more intense in both infected groups (**Figures 4C, E** - arrowheads).

Throughout all interaction periods in both models, Rab5 appears to be primarily recruited during the invasion process of *L. amazonensis* into murine fibroblasts (5h), while actin plays a more secondary role. In contrast, in the *L. braziliensis*-infected group, actin appears to be more actively involved in the parasite’s entry, particularly at the longest interaction time (24 hours).

## 4 Discussion

This study focused on the early stages of Leishmania infection in murine fibroblasts by different parasite species comparing different parasite species, interaction times, and 2D versus 3D culture models to better understand the factors influencing host-parasite interaction. We conducted qualitative and quantitative analyses to evaluate parasite adhesion and internalization, focusing on actin cytoskeleton involvement and Rab5a recruitment, an early endosome marker.

Our results show that parasite adhesion varies depending on the experimental model, infection time, and Leishmania species. In the 3D model, initial adhesion was lower than in the 2D model but increased progressively over time. This may be due to the structural complexity of the 3D environment where parasite migration requires prior degradation of the extracellular matrix (ECM) to reach host cells, making adhesion more gradual and time-dependent (Petropolis et al., 2014). Notably, after 24 hours of interaction, *L. braziliensis* (Lb) adhered more efficiently in the 3D model than *L. amazonensis* (La). This could reflect the greater migratory capacity of Lb promastigotes, previously described by our group (Costa et al., 2023) and may be related to the more invasive profile associated with this species often associated with the mucocutaneous leishmaniasis (Alves-Ferreira et al., 2015). This difference in behavior may also reflect distinct evolutionary invasion strategies, where *L. braziliensis*, with its greater motility and adhesion capacity, prioritizes faster and more active internalization routes, while *L. amazonensis* may rely more on vesicular mechanisms and early interaction with endosomal compartments (Tschoeke et al., 2014; Heeren et al., 2024).

Regarding the internalization mechanisms, we observed that fibroblasts, despite being non-professional phagocytes, actively engage the actin cytoskeleton during parasite entry. Membrane protrusions resembling filopodia and lamellipodia were detected near the sites of parasite invasion, in both 2D and 3D models. These structures are typically associated with actin remodeling and suggest the involvement of processes like non-professional phagocytosis or micropinocytosis (Mylvaganam et al., 2021). In contrast, previous work in the 2D model showed *L. amazonensis* can infect fibroblast independently of actin activity (Cavalcante-Costa et al., 2019)).

In professional phagocytic cells, such as macrophages, the role of actin during *Leishmania* infection is well established. In 2D models, parasite entry involves actin-rich structures like ruffles (Ramos et al., 2014; Dixit et al., 2021), and in 3D models, infection leads to enhanced expression of actin-regulation proteins associated with filopodia and lamellipodia (Luz et al., 2023). Together, these findings support the idea that Leishmania exploits actin-dependent mechanisms to invade both phagocytic and non-phagocytic cells.

Our data suggest that *L. amazonensis* utilizes actin-independent mechanisms of entry, supported by early Rab5a recruitment preceding cytoskeletal rearrangement. This may reflect clathrin-mediated or macropinocytosis-like pathways that facilitate early endosomal trafficking (Cavalcante-Costa et al., 2019; Rink et al., 2005; Ohashi et al., 2011). In contrast, *L. braziliensis* internalization is more dependent on actin engagement, possibly enabling faster invasion but triggering stronger host responses. This early recruitment of Rab5a indicates activation of typical endocytic pathways (Verma et al., 2017), suggesting that *L. amazonensis* may exploit pathways that evade immune detection or interfere less with host cell structure. The observed difference compared to *L. braziliensis* might therefore reflect not only the choice of entry route but also distinct strategies for intracellular persistence (Lecoeur et al., 2022). Further investigation is needed to characterize the post-entry events in fibroblasts, including parasite viability, replication, and intracellular trafficking, to clarify their role in pathogenesis.

Despite the valuable insights provided by this study, it is important to note that the current models, especially the 3D ones, do not fully recapitulate the complexity of *in vivo* conditions. Nevertheless, 3D models offer significant advantages in capturing aspects of host-parasite dynamics that are absent in 2D monolayers (Guo, 2020; Bassi et al., 2021). The presence of extracellular matrix components alters cellular tension, receptor localization, and endocytic profiles, which collectively impact the efficiency and route of parasite entry (Gaji et al., 2013; Alimohamadi and Rangamani, 2023). These biomechanical and biochemical cues likely play a role in determining the dominant internalization pathway used by each species.

Our findings indicate that *Leishmania* is capable of manipulating host cell machinery involved in actin cytoskeleton remodeling. The regulation could occur through molecular regulators such as Rho GTPases, including Cdc42 and Rac1 (Hoppe and Swanson, 2004; Tomasevic et al., 2007). Additionally, the parasite appears to modulate vesicular trafficking pathways, as evidenced by the recruitment of Rab5. This process could involve the participation of proteins such as PI3K (class III) and EEA1, which contribute to the generation and maturation of early endosomes (Omotade and Roy, 2019). However, the precise mechanisms by which *Leishmania* modulates these molecular regulators in 3D cell culture models remain unclear, highlighting the need for further investigations. Furthermore, the short duration of infection assessed in this study may not fully capture the dynamics of long-term immune responses or the strategies employed by the parasite to persist and survive during chronic infection.

Our findings align with previous studies indicating that protozoa parasites, including Leishmania, can employ multiple and sometimes complementary invasion mechanisms depending on the species and host cell context (Valigurová and Kolářová, 2023). This supports the notion that species-specific biological characteristics may influence differential host cell entry mechanisms, which could be linked to the clinical outcomes associated with each species (Silveira et al., 2024). In line with this, we observed distinct infection strategies between *L. amazonensis* and *L. braziliensis*, which may be crucial in understanding their varying pathogenic profiles (Silveira et al., 2009).

The mucocutaneous potential of *L. braziliensis* may be partly explained by its rapid and actin-dependent invasion, possibly triggering stronger local inflammatory responses and deeper tissue infiltration (Alves-Ferreira et al., 2015). On the other hand, *L. amazonensis*, often linked to chronic cutaneous lesions, may benefit from more discreet entry routes that help sustain long-term persistence with limited host damage, in line with its intracellular trafficking preference (Martinez and Petersen, 2014). Although our study focused on elucidating the early stages of *Leishmania* infection, it was limited to the use of promastigote forms, as these are the forms that interact with host cells. In contrast, amastigotes are associated with the persistence and amplification of infection and play a more critical role in later stages of pathogenesis. Therefore, future studies are warranted to investigate the behavior and host interactions of amastigotes, particularly in the context of persistent infection.

While early infection stages demonstrated similar patterns across both species, extended interaction periods could reveal divergent strategies, emphasizing the importance of time in evaluating *Leishmania* infection. The data obtained from 3D models are still limited, underscoring the need for more comprehensive studies. In particular, the three-dimensional extracellular matrix in these models plays a key role in influencing cell dynamics, affecting factors such as cytoskeletal organization, receptor distribution, and intracellular trafficking post-invasion (Yamada et al., 2019; Doyle et al., 2022). This contributes to the observed differences between the 2D and 3D models and provides a closer approximation of *in vivo* conditions, offering a more accurate representation of *Leishmania*–host interactions in non-phagocytic cells.

The identification of species-specific endocytic and cytoskeletal dependencies in *L. amazonensis* and *L. braziliensis* highlights promising therapeutic opportunities. Pharmacological inhibition of key components such as Rab5a or actin polymerization - using agents like wortmannin, cytochalasin D, or dynasore - may disrupt parasite entry and early survival (Maganto-Garcia et al., 2008; Bonifácio et al., 2022). Targeting the predominant invasion route of each species could help reduce parasite load while minimizing off-target effects on host cells.

In conclusion, our results demonstrate that different *Leishmania* species employ distinct invasion strategies, shaped by both the experimental and the host cell type. Despite these differences, a common feature is the parasite’s ability to manipulate host cell machinery, particularly through actin cytoskeleton remodeling. These findings reinforce the complexity of host-parasite interactions and highlight the parasite’s adaptative capacity to diverse microenvironments. Furthermore, our data support the idea that fibroblasts, although non-professional phagocytes, can serve as host cells for both *L. amazonesis* and *L. braziliensis*. This reinforces the notion that *Leishmania* can exploit alternative pathways for entry and survival in non-phagocytic cells, with the specific invasion strategy influenced by the parasite species, even though they do not have the machinery available in professional phagocytes such as macrophages.

Future studies involving infection periods and more complex three-dimensional models are essential to further elucidate the molecular mechanisms underlying these processes. A deeper understanding of these mechanisms will be critical for the development of targeted therapeutic strategies aimed at blocking or interfering with the early stages of infection, contributing to more effective control of leishmaniasis.

## Data Availability

The original contributions presented in the study are included in the article/**Supplementary Material**. Further inquiries can be directed to the corresponding author.
