# Supplementary material for: Impact of 2D versus 3D fibroblast models on Leishmania species invasion in vitro: Rab5 dynamics and actin activity in initial infection
Source: Front Cell Infect Microbiol. 2025 Aug 18;15:1654654. doi: 10.3389/fcimb.2025.1654654 (PMC12399602; doi:10.3389/fcimb.2025.1654654)
Supplement: Supplementary file 3 [file Table1.docx]

**Supplementary materials**

The Rab5 recruitment in the process of *Leishmania amazonensis* invasion in fibroblasts in 3D model at 18 hours and 24 hours, respectively.

Supplementary video 1: FIB+LA RAB5 18H

Supplementary video 2: FIB+LA RAB5 24H
